# Facing your fears in schools: using the ADIS/ASA to characterize anxiety and intervention outcomes in students with autism or suspected autism

**DOI:** 10.3389/fpsyt.2025.1569435

**Published:** 2025-07-23

**Authors:** Judy Reaven, Kelly T. Cosgrove, Ainsley Losh, Sarah Nickles, Connor M. Kerns, Katherine Pickard, Audrey Blakeley-Smith, Lisa Hayutin, Allison T. Meyer, Caitlin Middleton, Nuri M. Reyes, Richard E. Boles

**Affiliations:** ^1^ JFK Partners, University of Colorado Anschutz Medical Campus, Aurora, CO, United States; ^2^ Department of Psychiatry, School of Medicine, University of Colorado Anschutz Medical Campus, Aurora, CO, United States; ^3^ Department of Pediatrics, Children’s Hospital Colorado, Aurora, CO, United States; ^4^ Department of Psychology, The University of British Columbia, Vancouver, BC, Canada; ^5^ Department of Pediatrics, Emory University School of Medicine, Atlanta, GA, United States

**Keywords:** autism, anxiety, school-based intervention, cognitive behavioral therapy (CBT), anxiety disorders interview schedule -autism spectrum addendum (ADIS/ASA), implementation

## Abstract

**Background:**

Autistic youth are at higher risk of developing anxiety compared to their peers, with as many as 40% experiencing clinical anxiety. Emerging research suggests that these rates are an underestimate as distinct presentations of anxiety (e.g., fear of change, idiosyncratic fears) are often not recognized. CBT is a well-established approach for managing anxiety in autistic youth, but many have difficulty accessing these interventions. School-based CBT programs, like Facing Your Fears in Schools (FYF-S), have shown effectiveness in reducing anxiety in autistic students and may increase access to care. The Anxiety and Related Disorders Interview Schedule for DSM-5 with Autism Spectrum Addendum is a semi-structured interview that captures both DSM-5 and distinct presentations of anxiety. This study aimed to: 1) characterize a subsample of students with autism or suspected autism and anxiety and 2) examine effectiveness of FYF-S using the ADIS/ASA.

**Methods:**

This study utilized a subsample of students (*N*=37; ages 7-14) from a larger Type 1 hybrid-effectiveness trial who had either autism or suspected autism. Students were randomized to either FYF-S or Usual Care (UC). Caregivers completed the ADIS/ASA at baseline and post-intervention. The ADIS/ASA was administered by clinicians rigorously trained to reliability and masked to condition.

**Results:**

Students had both DSM-5 and distinct anxiety diagnoses at Time 1. Further, students in FYF-S demonstrated significant reductions in anxiety compared to UC, as evidenced by fewer anxiety diagnoses overall and significant improvement in total anxiety.

**Conclusion:**

This is the first school-based study using the ADIS/ASA to characterize anxiety and measure outcomes in autistic students. Overall, results indicate that FYF-S may be a promising school-based intervention for autistic youth.

**Clinical trial registration:**

https://clinicaltrials.gov/study/NCT03685474, identifier NCT03685474.

## 1 Introduction

Children’s mental health has been of growing concern in recent years, as it has been estimated that between 13-20% of children in the United States experience a mental health concern every year (1). Anxiety is among the most commonly experienced symptoms for children under the age of 18 with rates of approximately 8% reported according to the National Survey of Children’s Health (a population-based sample) (1). Notably, autistic children are at risk for experiencing higher rates of anxiety, compared to non-autistic youth (2, 3). Previous estimates suggest up to 80% of autistic children experience at least subclinical levels of anxiety (3) and nearly 40% meet criteria for at least one clinical anxiety diagnosis (4). However, commonly reported prevalence rates may underestimate the presence of anxiety in autism because they do not capture distinct anxiety presentations in autism (e.g., fear of change, fears related to sensory stimuli, perceived inability to engage in focused interests, and fears related to social unpredictability; (5–7)).

Importantly, anxiety can interfere significantly across contexts and settings, including at home and in school (8). Poor academic achievement and adaptive outcomes, along with reduced engagement in academic and non-academic activities are notable impacts for autistic students (8). Common school-based fears may include loud noises (e.g., fire alarms, intercom announcements), changes in the school routine, making mistakes, hesitation in starting new tasks and numerous social situations (5, 7).

Cognitive Behavioral Therapy (CBT) is the gold standard for the treatment of anxiety and other mood disorders for non-autistic individuals (9, 10). CBT aims to replace negative cognitive constructs about the world, self, or future with more flexible and adaptive cognitions (11). Common strategies employed in CBT for anxiety include psychoeducational strategies such as cognitive restructuring to identify and challenge negative thoughts and somatic management (e.g., relaxation and breathwork), as well as graded exposure practice to anxiety provoking situations (12).

Over the past 15 years, randomized trials have examined the efficacy/effectiveness of adapted CBT for the treatment of anxiety in autistic youth (13, 14). Common adaptations have included the use of written materials, role-playing, caregiver involvement, and delivering psychoeducation in smaller, more manageable components (12, 15, 16). Several meta-analyses indicate that both individual and group-delivered CBT resulted in significant reductions in anxiety symptoms for autistic youth according to multi-informant report (e.g., caregiver, clinician, and child/self-report; (12, 14, 17)), signaling the effectiveness of CBT approaches.

Despite the growing body of evidence supporting CBT interventions for anxiety in autism, families face challenges in accessing these services. Barriers include long waitlists, limited providers with knowledge of mental health *and* autism, logistical strains (e.g., transportation, childcare), and difficulties navigating the healthcare system broadly (e.g., cost, insurance coverage, healthcare literacy) (18). Notably, these barriers disproportionately impact families from historically marginalized communities, who are also underrepresented in research studies evaluating the effectiveness of mental health interventions for autistic youth (19–21).

Schools may be well-positioned to more equitably deliver CBT to autistic youth in school settings, as they may alleviate many of these barriers (22–24). School-based mental health support may reduce the burden on caregivers to navigate the complex healthcare system, provide transportation, weigh the potential cost of missed work, and arrange for childcare, if needed. Indeed, previous research suggests that adolescents with public insurance, from lower socioeconomic households, and from minoritized racial and ethnic backgrounds are more likely than their peers to receive mental health interventions *only* in educational settings (22). Thus, schools have the potential to reach autistic youth with mental health needs who may not otherwise have access to care.

Recent research has begun to investigate the effectiveness of CBT interventions for autistic students with anxiety in school-based settings, and preliminary findings are encouraging (25–27). One school-based CBT program developed specifically for autistic youth with anxiety is *Facing Your Fears in Schools* (*FYF-S*; (28)). FYF-S is a manualized intervention comprised of psychoeducation and graded exposure components delivered in small groups by trained interdisciplinary school providers. The school-based program was adapted from the Facing Your Fears clinic-based program (16, 29) and has demonstrated effectiveness in reducing anxiety in autistic students in pilot studies and a cluster randomized trial (28, 30, 31).

Intervention outcomes in schools have primarily been measured using standardized rating scales, such as the *Screen for Anxiety and Related Emotional Disorders (SCARED*; (32)), *Spence Children’s Anxiety Scale (SCAS (* 33 *);*), *Multidimensional Anxiety Scale for Children – 2nd Edition (MASC-2;* (34)), or *School Anxiety Scale (SAS*; (35)). These rating scales have many advantages as they are efficient and easy to administer in school settings, have objective clinical cut-off scores, are supported by validity and reliability evidence, and result in quantitative data for ease of comparison across groups and timepoints. However, most were not developed specifically for autistic youth, and may have reduced screening accuracy or different factor structures in this population, as they may not capture distinct anxiety presentations (36, 37). Some school-based CBT research has integrated semi-structured interviews in addition to rating scales to obtain qualitative information such as children’s experiences navigating challenging situations at school, parents’ perspectives on children’s use of coping strategies, and school providers’ feedback about interventions (24, 25). However, no known standardized, semi-structured interviews have been used to more completely understand the nature of anxiety in autistic youth in the context of school-based intervention.

Fortunately, systematic efforts have been made to further understand anxiety in autistic youth. Kerns and colleagues (5) developed the Autism Spectrum Addendum (ASA) for the Anxiety Disorders Interview Schedule – IV – Parent Interview (ADIS-P; (38)), to understand the extent to which autistic youth experience DSM-5 diagnoses of anxiety and distinct presentations of anxiety. Distinct presentations of anxiety are defined as impairing fears that do not conform to traditional DSM-5 categories (5). Examples of distinct presentations of anxiety include idiosyncratic phobias (e.g., fears of men with beards), special interest fears (i.e. fear of not being able to engage in focused interests), fear of change (i.e., intense anxiety about small changes in environment or routine), and other social fears (i.e., fear of social situations and interaction *without* fear of negative evaluation or embarrassment).

In one of the most comprehensive studies to date, caregivers of autistic youth (*n*=75) and non-autistic youth (*n*=52) between the ages of 9–13 of varying intellectual ability were administered the ADIS/ASA to obtain information about the presence of DSM-5 and distinct presentations of anxiety in each group (6). Results indicated that autistic youth were much more likely than non-autistic youth to meet criteria for a DSM-5 anxiety diagnosis (21% vs 8%). The most common DSM-5 anxiety diagnoses for autistic youth included specific phobia, generalized anxiety, separation anxiety, and social anxiety. Nearly a third (31%) of autistic children in this sample had both DSM-5 and distinct anxiety presentations. Twenty-one percent (21%) had DSM-specific anxiety only, while 17% had distinct anxiety presentations only, compared with none in the non-autistic comparison group. The most commonly endorsed distinct fear was “fear of change.” These results suggest that anxiety measures not designed for autistic youth may fail to capture the full range of anxious symptoms and diagnoses.

The ADIS/ASA has now been used as an eligibility measure to confirm clinical anxiety in autistic participants (39, 40), to characterize anxiety prevalence and characteristics (41, 42), and as an outcome measure (43–45). Notably, most research employing the ADIS/ASA has occurred in university and/or specialty clinic settings. As intervention trials move from these clinics to community settings (e.g., schools), it will be important to more fully understand the presentation of anxiety in autistic students, both at baseline (Time 1) and post-intervention/post-usual care (UC) (Time 2). This is particularly important given that many youth are unable to access university-based research studies (20).

The purpose of the current study is to examine a subsample of students with autism or suspected autism and anxiety who were enrolled in a Type 1 hybrid-effectiveness trial examining the effectiveness of FYF-S using the ADIS/ASA. Previous intervention outcomes have been reported on the full sample (*n*=81) using various objective measures (28). The aims of the current study are to: 1) characterize a subsample of students with autism or suspected autism and anxiety at Time 1 using the ADIS/ASA; and 2) examine outcomes of the student participants randomized to either FYF-S or UC using the ADIS/ASA. It was hypothesized that participants in this sample will meet criteria for both DSM-5 and impairing, distinct presentations of anxiety at Time 1. Additionally, it was hypothesized that participants randomized to FYF-S will demonstrate significant improvements in anxiety compared to students randomized to UC at Time 2.

## 2 Methods

### 2.1 Participants

The present study involved 37 elementary and middle school students aged 7–14 with autism or suspected autism and their caregivers from three school districts in Colorado. All participants were enrolled in a larger cluster randomized trial investigating the effectiveness of FYF-S compared to UC (28). Participants across the three school districts were given the opportunity to complete the ADIS/ASA. The ADIS/ASA interview was offered as an optional study procedure (given the additional time it takes to participate in the interview). Caregivers who completed the interview were from each of the districts and were provided with additional compensation ($25). Analyses of demographic data comparing participants who completed the primary measure for the present study (ADIS/ASA) (*n*=37) to other participants (*n*=81) in the larger trial revealed no significant differences (*p* > 0.05) for child and parent age, sex of child, language spoken at home, highest level of education, household income, marriage status, prior diagnoses (i.e., autism, attention deficit/hyperactivity disorder (ADHD), anxiety, and depression), although participants were more likely to have completed the ADIS/ASA if they identified as white (χ^2^ = 12.52, *p* < 0.001) and non-Hispanic (χ^2^ = 5.17, *p* < 0.05). Inclusion criteria for the parent study were as follows: caregiver-reported difficulties in social communication using the Social Responsiveness Scale, Second Edition (SRS-2; (46)) and clinically significant levels of anxiety. For the SRS-2, a total T score above 60 was used as a cutoff, reflecting the presence of at least mild autistic symptoms per previous research (31). To meet criteria for clinically significant anxiety, students had at least one clinically elevated score on any of the following anxiety measures: The SCARED (Child or Parent Report) (32), Parent Rated Anxiety Scale for Autism Spectrum Disorder (PRAS-ASD) (47), or the SAS–Teacher Report (35). Students also had an Individualized Education Program (IEP) so that FYF-S could be delivered as part of their IEP services. Exclusion criteria for the study included having a known intellectual disability or significant mental health or behavioral challenges (e.g., inability to work in a small group, significant physical aggression, psychiatric hospitalization) that could hinder participation in the group program. Ninety-nine students were screened for the parent study, and 18 of these were either excluded (*n*=5; did not meet inclusion criteria) or were not enrolled due to lack of interest in study participation (*n*=13). Of the 81 families enrolled in the study, 37 agreed to complete the ADIS/ASA at Time 1 (*n*=20 FYF-S; *n*=17 UC) and comprise the sample for the present analysis. Demographic details for this sample are listed in **Table 1**.

**Table 1:** Baseline participant demographics (*N*=37).

Variable	Entire Sample (*N*=37)	FYF-S (*n*=20)	UC (*n*=17)	χ2/*t* (df)	*p*
Age in years, mean (SD)	10.49 (2.04)	11.28 (2.20)	10.66 (1.75)	0.94 (35)	0.35
Sex				0.29 (1)	0.59
Male	29 (78.4%)	15 (75%)	14 (82%)		
Female	8 (21.6%)	5 (25%)	3 (18%)		
Ethnicity				0.05 (1)	0.83
Hispanic or Latino	6 (16.2%)	3 (15%)	3 (18%)		
Non-Hispanic or Latino	31 (83.8%)	17 (85%)	14 (82%)		
Race^1^					
White	34 (91.9%)	18 (90%)	16 (94%)	0.21 (1)	0.65
American Indian	1 (2.7%)	1 (5%)	0 (0%)	0.87 (1)	0.35
Asian	2 (5.4%)	1 (5%)	1 (6%)	0.01 (1)	0.91
Black	5 (13.5%)	3 (15%)	2 (12%0	0.08 (1)	0.77
Native Hawaiian/Pacific Islander	1 (2.7%)	1 (5%)	0 (0%)	0.87 (1)	0.35
Other/More than one race	1 (2.7%)	0 (0%)	1 (6%)	1.21 (1)	0.27
Primary language				0.87 (1)	0.35
English	36 (97.3%)	19 (95%)	17 (100%)		
Other	1 (2.7%)	1 (5%)	0 (0%)		
Consenting caregiver sex				1.05 (1)	0.31
Male	7 (18.9%)	5 (25%)	2 (12%)		
Female	30 (81.1%)	15 (75%)	15 (88%)		
Consenting caregiver employed				1.80 (1)	0.18
No	8 (21.6%)	6 (30%)	2 (12%)		
Yes	29 (78.4%)	14 (70%)	15 (88%)		
Consenting caregiver education				4.45 (6)	0.62
Some high school	1 (2.7%)	1 (5%)	0 (0%)		
High school graduate	6 (16.2%)	4 (20%)	2 (12%)		
Some college, no degree	10 (27.0%)	5 (25%)	5 (29%)		
Associate’s degree	2 (5.4%)	0 (0%)	2 (12%)		
Bachelor’s degree	10 (27.0%	5 (25%)	5 (29%)		
Some graduate training/	4 (10.8%)	2 (10%)	2 (12%)		
Master’s degree					
Professional degree	4 (10.8%)	3 (15%)	1 (6%)		
Annual household income				5.47 (9)	0.79
$5,000-24,999	3 (8.1%)	1 (10%)	2 (12%)		
$25,000-49,999	5 (13.5%)	3 (15%)	2 (12%)		
$50,000-74,999	7 (18.9%)	4 (20%)	3 (18%)		
$75,000-99,999	4 (10.8%)	3 (15%)	1 (6%)		
$100,000+	12 (32.4%)	6 (30%)	6 (35%)		
Unknown/not reported	5 (13.5%)	2 (10%)	3 (18%)		

^1^participants could select multiple responses.

### 2.2 Study design

This study is a secondary data analysis of a cluster randomized trial of FYF-S. See Reaven and colleagues (28) for a comprehensive description of the procedures for the parent trial. The study protocol was approved by the Colorado Multiple Institutional Review Board and the research review boards of each participating school district. Study procedures were conducted in accordance with the Declaration of Helsinki. The study is registered on clinicaltrials.gov (identifier: NCT03685474). Data were collected and managed using Research Electronic Data Capture (REDCap) tools hosted at the University of Colorado Denver (48). Participants were compensated for their time.

Three school districts in Colorado approved the study procedures and acted as study sites. School teams were comprised of Interdisciplinary School Providers (ISPs; e.g., school psychologists, speech/language pathologists, special educators, occupational therapists) from each district who were recruited to participate in the study. All ISPs provided written informed consent to participate and received an initial three-hour training about autism and anxiety. Then, the school teams were randomized to either FYF-S or UC. ISPs in the FYF-S condition received an additional nine-hour training on the intervention and were given program materials. Following training, all ISPs identified students in their schools who may be appropriate for the study and obtained permission from the students’ caregivers to be contacted by the study team.

Interested caregivers provided written informed consent for their students to participate in the study. Caregivers were then asked to complete the aforementioned qualification measures. Students provided assent to participate in the study and completed their own qualification and baseline measures during in-person study visits at their schools. For caregivers who agreed to complete the ADIS/ASA, the interview was administered in-person or virtually by doctoral-level psychologists who were rigorously trained to reliability by the interview developer (CMK) and masked to intervention condition. All ADIS/ASA interviewers were required to attend a training on the measure led by CMK, including co-scoring several interviews together. Following formal training, each interviewer submitted additional scores for three recorded ADIS/ASA interviews and submitted their own recording for a reliability check (i.e., reliability was defined as administering the interview with fidelity and achieving 100% diagnostic agreement and no more than 1 point deviation on Clinical Severity Ratings [see description below] for each diagnosis given with CMK.

School teams randomized to FYF-S began intervention sessions after Time 1 data collection, while students in the UC condition continued their typical school-based and community services. Following the intervention time period, all caregivers were sent a link to complete follow-up measures via REDCap. Those who completed the ADIS/ASA at Time 1 were invited to complete this interview again. All follow-up measures were completed within two to six weeks after the intervention period.

### 2.3 Intervention

#### 2.3.1 Facing your fears in schools

FYF-S is a manualized CBT intervention specifically designed for autistic students and students with similar social and communication differences who experience anxiety. This school-based program, adapted from the clinic-based FYF program (16, 29, 49), consists of 12, 40-minute sessions. The first half of the program focuses on psychoeducation and skill-building, while the second half employs graded exposure techniques to help students gradually face fears. Ideally, FYF-S is delivered by a minimum of two ISPs in small groups of two to four students. For the parent cluster randomized control trial, ISPs were trained in FYF-S using a train-the-trainer approach. Reaven and colleagues (28) demonstrated that the ISPs were able to successfully implement FYF-S with high fidelity and adherence.

#### 2.3.2 Usual care

Students in UC were informed that they could continue to engage in any school- or community-based interventions they were receiving prior to the study. Detailed information regarding these interventions was not collected for the larger study.

### 2.4 Measures

Student and caregiver demographic data (e.g., age, grade, sex, race/ethnicity) were collected via caregiver report on a brief questionnaire. Primary outcome measures have already been reported for the parent trial (see (28)). The present study focuses on secondary outcomes derived from the ADIS/ASA.

Anxiety and Related Disorders Interview Schedule for DSM-5 with Autism Spectrum Addendum (ADIS/ASA; (50, 51)) is a clinician-administered, semi-structured parent interview designed to assess anxiety in autistic youth, which takes 60–120 minutes to administer (5). This measure was developed by Kerns and colleagues (5) as an extension of the original ADIS (38). As noted above, this interview assesses DSM-5 anxiety disorders, including generalized anxiety disorder (GAD), specific phobia, social anxiety disorder, separation anxiety disorder, and obsessive-compulsive disorder (OCD). It also measures distinct presentations of anxiety that are experienced by autistic youth (i.e., impairing fears that do not conform to traditional DSM-5 categories, including fear of change, special interest fears, idiosyncratic phobias, and other social fears). The ADIS/ASA also measures some non-anxiety-based diagnoses (e.g., ADHD, depression, oppositional defiant disorder) (5). Previous research has shown that the ADIS/ASA demonstrates good psychometric properties, including 2-week retest reliability, high interrater reliability and discriminant validity for traditional and distinct anxiety symptoms as well as concurrent validity with other traditional anxiety measures (5, 52, 53). Forty-three percent (43%) (*n*=15) of the ADIS/ASA interviews conducted for the present study were randomly selected equally from Time 1 and Time 2 and co-scored for reliability of all diagnoses (“Yes/No”) and results of Cohen’s Kappa tests for reliability showed a range from .86 to 1.00 and an overall Kappa mean (sd) of .97 (.06), indicating almost perfect level of agreement (54).

The ADIS/ASA provides Clinical Severity Ratings (CSRs) for each diagnosis that can range from 0 (not impairing) to 8 (debilitating), based on examples of interference provided by parents across various domains (e.g. home, school, social/peer relations). A CSR of 4 or higher indicates the presence of a clinical diagnosis (5). Thus, CSRs can be used to both confirm the existence of a diagnosis and describe the severity of the symptoms associated with a diagnosis (even if these do not meet the threshold for clinical diagnosis). The diagnosis with the highest CSR at Time 1 was identified as the “primary diagnosis.” In the event that there was more than one diagnosis with the same CSR score, the clinician queried further and asked the caregiver which diagnosis they perceived to be the primary diagnosis. The primary diagnosis could be any anxiety diagnosis on the measure (i.e., both DSM-5 and distinct).

The ADIS/ASA was also used to inform Clinical Global Impressions Scale - Improvement ratings (CGIS-I; (55)). CGIS-I ratings were completed by masked evaluators at Time 2. Scores range from 1 (very much improved) to 7 (very much worse). Four CGIS-I scores were calculated. First, a Primary CGIS-I was determined based on the amount of change in primary diagnosis CSR from Time 1 to Time 2. This approach is similar to other published work with the ADIS/ASA (39, 43, 50). Next, we calculated a Total Anxiety Composite CGIS-I for *all* anxiety diagnoses with the exception of specific phobia (e.g., GAD, social anxiety, separation anxiety, OCD, fear of change, other social fear, special interest fear, negative reaction to change). This was calculated based on the average change in CSR from Time 1 to Time 2 across *all* identified anxiety diagnoses on the ADIS/ASA. Specific phobia was one of the most common diagnoses endorsed in the current sample, and many participants had multiple phobias. For this reason, we decided to examine a separate composite score for specific phobias (Phobia Composite CGIS-I). A similar composite was also created for all non-anxiety diagnoses (ADHD, major depressive disorder, oppositional defiant disorder, and post-traumatic stress disorder; Non-Anxiety Composite CGIS-I). As with the Anxiety Composite, the Phobia Composite CGIS-I and Non-Anxiety Composite CGIS-I were calculated based on the average change in CSR for all diagnoses identified in their respective categories.

### 2.5 Data analytic plan

Descriptive statistics were used to summarize information about participant demographics across the FYF-S and UC groups at Time 1. Chi square analyses and t-tests were used to test for significant group differences with phi coefficients (56), where associations from 0 to < 0.10 are *negligible*, 0.10 to < 0.20 are *weak*, 0.20 to < 0.40 are *moderate*, 0.40 to < 0.60 are *relatively strong*, 0.60 to < 0.80 are *strong*, and 0.80 to 1.00 are *very strong* (56). To address the first aim of this study (i.e., characterizing the diagnostic picture of the school-based sample of autistic youth at Time 1), we calculated the frequencies of each clinical diagnosis identified during the ADIS/ASA interview (i.e., those with CSRs ≥ 4). This included DSM-5 anxiety diagnoses, distinct anxiety presentations, and non-anxiety-based diagnoses. Of note, we combined social anxiety with other social fear and fear of change with negative reaction to change (i.e. distress following, but not in anticipation of changes) in these analyses for parsimony due to the small sample size and because of the conceptual similarities, although this is not recommended by the authors of the ADIS/ASA measure^^1^^.

To address the second aim of examining intervention-related changes in anxiety as measured by the ADIS/ASA, several approaches were taken. First, we examined changes in the frequency of the DSM-5 anxiety diagnoses across time for each group using chi-square analyses, including estimate of phi correlations (strength of associations among two binary variables). Next, we conducted generalized linear mixed models (GLMMs) to estimate treatment effects of FYF-S compared to UC on CSRs and mean number of diagnoses. These models included group (FYF-S vs UC) and time (Time 1 vs Time 2) as fixed effects and subject as a correlated random effect to account for individual level heterogeneity that may be correlated to time-based predictor variables. *Post-hoc* chi-square analyses were conducted for any significant GLMM results. Lastly, we conducted independent t-tests to compare CGIS-I scores (Primary, Total Anxiety Composite, Specific Phobia Composite, and Non-Anxiety Composite) between FYF-S and UC.

## 3 Results

### 3.1 Characterization of sample

Child participants were an average age of 10.49 ± 2.0 years (range: 7-14) across both groups. The majority were male (78%), non-Hispanic (84%), and white (92%). The majority of caregivers identified as female (81%) and reported some college or higher education attainment (81%). With regard to annual household income, approximately 22% reported incomes below $50,000, 19% reported incomes between $50,000-$74,999 and 43% reported income above $75,000. Chi-square or independent t-test comparisons between the groups showed no significant baseline differences across all demographic variables, *p* > 0.05. See **Table 1** for complete demographic details.

#### 3.1.1 Frequency of diagnoses

**Figure 1:**
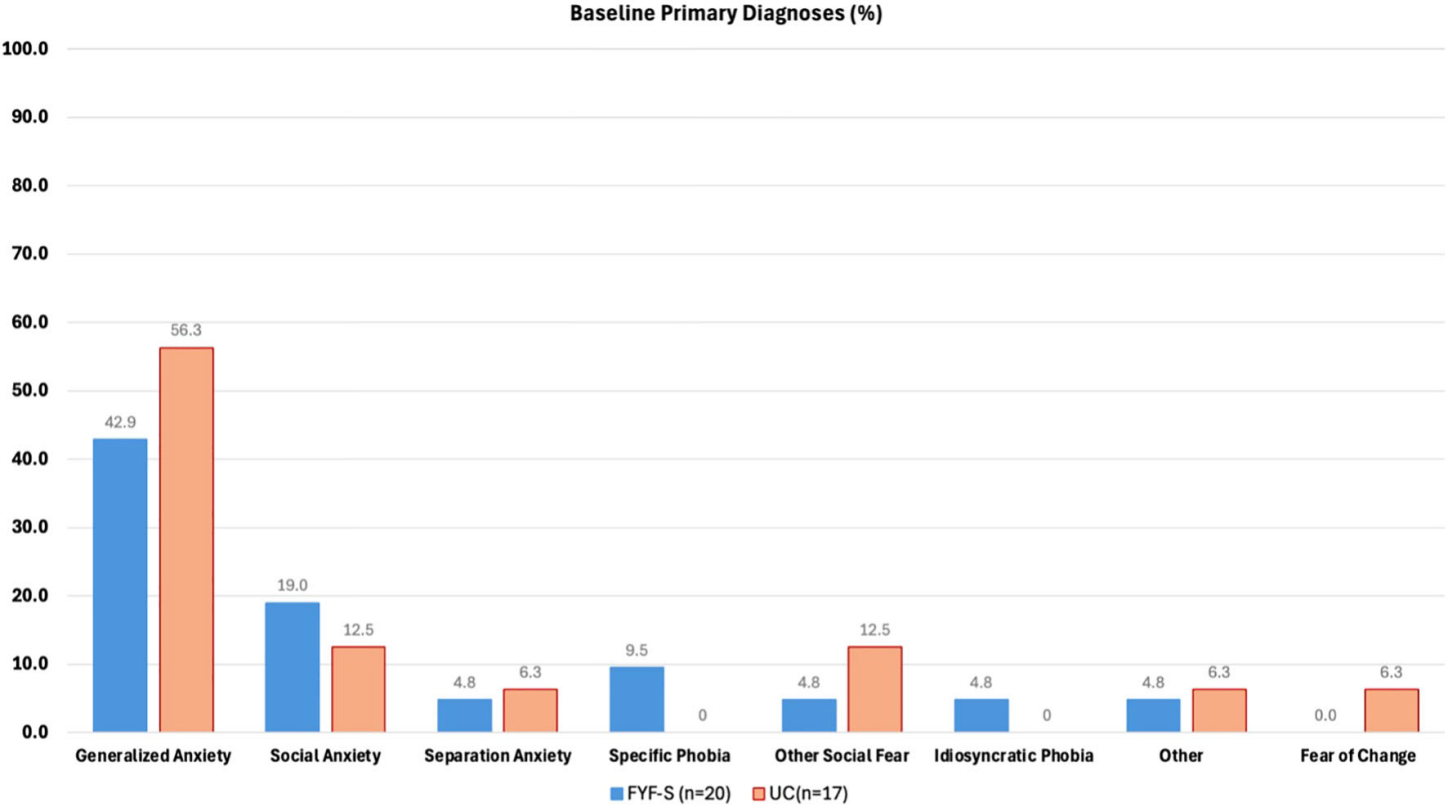
Frequency of primary diagnosis for Facing Your Fears in Schools (FYF-S) and Usual Care (UC) at Time 1.

The frequency of DSM-5 diagnoses for the total sample and by those randomized to FYF-S or UC are presented for Time 1 and Time 2 in **Table 2**. There were no differences between conditions at Time 1 for most diagnoses, although participants assigned to FYF-S presented with significantly more specific phobia diagnoses compared to participants randomized to UC (84% vs 44%). The most common DSM-5 anxiety diagnoses for the entire sample at Time 1 included GAD (68%), specific phobia (66%), and social anxiety disorder (46%). Of note, the average (SD/range) number of phobias for all participants was 1.67 (1.59/0-4) at Time 1. Specific phobias endorsed across timepoints were comprised of 14 unique phobias. Common phobias that were endorsed included loud noises, blood/injections/needles, animals/insects, and the dark. Among all participants, six idiosyncratic phobias were endorsed across timepoints, such as fears of burping and of the home hallway. Distinct anxiety diagnoses most often included negative reaction to change (38%) and fear of change (30%). Among the total sample at Time 1, 22% met criteria for a DSM-5 diagnosis only, none of the participants had solely a distinct presentation of anxiety, and 76% had both DSM-5 and distinct anxiety diagnoses. Among other non-anxiety related DSM-5 diagnoses, ADHD-Combined type was most often diagnosed (57%). See **Table 2** for more information. There were no group significant differences in mean number of DSM-5 diagnoses at T1 (independent t-test *p* < 0.05). Primary diagnoses were similar across FYF-S and UC, in which the most common primary diagnosis was GAD (42.9% and 56.3%), followed by social anxiety (19.0% and 12.5%), respectively. All other diagnoses were at or below 12.5% and are shown in **Figure 1**.

**Table 2:** ADIS/ASA diagnoses in the FYF-S and UC groups across time points.

Diagnosis	Time 1			Time 2		
	Entire Sample (*N*=37)	FYF-S (*n*=20)	UC (*n*=17)	Entire Sample (*N*=22)	FYF-S (*n*=11)	UC (*n*=11)
DSM-5 Anxiety Diagnoses						
DSM-5 diagnosis only	22%	24%	19%	26%	17%	36%
Generalized Anxiety Disorder^2^	68%	62%	75%	61%	42%	82%
Specific Phobia^1,a^	66%	84%	44%	32%	27%	36%
Social Anxiety Disorder	46%	48%	44%	35%	33%	36%
Separation Anxiety Disorder	22%	19%	25%	22%	17%	27%
Obsessive Compulsive Disorder	3%	0%	6%	0%	0%	0%
Distinct Anxiety Diagnoses						
Distinct diagnosis only	0%	0%	0%	0%	0%	0%
Negative Reaction to Change	38%	29%	50%	30%	25%	36%
Fear of Change	30%	33%	25%	13%	8%	18%
Other Social Fear	22%	24%	19%	13%	8%	18%
Special Interest Fear	11%	14%	6%	30%	25%	36%
Idiosyncratic Phobia	5%	10%	0%	9%	8%	9%
Both DSM-5 and distinct diagnoses	76%	71%	81%	65%	67%	64%
Non-Anxiety Diagnoses						
Attention Deficit/Hyperactivity Disorder	57%	53%	63%	56%	58%	55%
Oppositional Defiant Disorder	11%	10%	13%	13%	17%	9%
Major Depressive Disorder	3%	0%	6%	9%	8%	9%
Posttraumatic Stress Disorder	3%	0%	6%	0%	0%	0%

#### 3.1.2 Diagnostic overlap and differences regarding GAD and fear of change

Phi correlations were conducted between GAD and fear of change, which showed GAD was significantly positively associated with fear of change, r_Φ_ = 0.32, *p* = 0.04, and considered a moderate association. Notably, being diagnosed with GAD did not always include a diagnosis of fear of change, in which FYF-S participants at Time 1 with GAD included 29% without a fear of change diagnosis. At Time 2 the percentage of those with GAD but not diagnosed with fear of change was 33%. The participants in UC showed a similar pattern in which 56% were diagnosed with GAD but not diagnosed with fear of change at Time 1. At Time 2 the percentage of those with GAD but not diagnosed with fear of change was 64%. By contrast, all participants with impairing fears of change were also diagnosed with GAD at Time 1 (33%) and Time 2 (8%), while UC participants at Time 1 diagnosed with fear of change (25%) included one less participant with diagnoses for both GAD and fear of change (19%). The percentage of fear of change for UC at Time 2 was the same as being diagnosed with both GAD and fear of change (18%).

### 3.2 Intervention outcomes

At Time 2, group differences in percentage of diagnoses were present for GAD (χ^2^ = 3.88, p <.049) _Φ_ = .41, a relatively strong effect), with 42% for FYF-S vs 82% for UC. Within FYF-S group differences across time showed significant decreases in rates of diagnoses for Specific Phobia (χ^2^ = 8.19, p <.004) Φ=.68, considered a strong effect), with 84% for Time 1 vs 27% for Time 2. Within UC group differences across time showed no significant differences in rates of diagnoses (*p*s > 0.05; **Table 2**).

#### 3.2.1 Specific phobia

The ADIS/ASA allows for multiple specific phobia diagnoses per participant, necessitating analysis of both diagnosis prevalence and average number of phobias. In FYF-S, participants had an average of 1.5 phobias (SD = 1.41, range 0-4) at Time 1 and 1.5 phobias (SD = 1.05, range 0-3) at Time 2. While the *average* remained constant, significantly fewer participants met criteria for *any* specific phobia diagnosis at Time 2 (*p* < 0.05, see **Table 2**). In UC, participants averaged 1.9 phobias (SD = 1.86, range 0-4) at Time 1 and 0.57 phobias (SD = 0.79, range 0-2) at Time 2, indicating a decrease in average phobias for those still meeting criteria. Notably, more participants in the FYF-S group lost the specific phobia diagnosis entirely compared to the UC group.

#### 3.2.2 Primary diagnosis CSR

Primary Diagnosis CSR showed a significant effect for Time (*F* (1, 28.22) = 21.79, *p* < 0.001); however, the Time by Group interaction was non-significant (*F* (1, 28.22) = 0.46, *p* = 0.50). Separate models for each group were explored to test individual change over time, in which FYF-S was significantly improved (*F* (1, 12.34) = 24.40, *p* < 0.001), with mean Primary Diagnosis CSR decreasing from 5.33 (.84) at Time 1 to 4.00 (1.55) at Time 2. Similarly, the model for UC showed significant improvement (*F* (1, 15.77) = 6.29, *p* < 0.023), with mean Primary Diagnosis CSR decreasing from 5.18 (.75) at Time 1 to 4.18 (1.54) at Time 2. Therefore, the CSRs for primary diagnoses improved for both groups, though with a larger effect in the FYF-S group (Cohen’s d = 1.49) compared to UC (Cohen’s d = 0.61). Further, there were no differences between condition with regard to resolution of primary diagnosis. Out of the 11 participants for each group, 4 participants from each group (36%) no longer met criteria for their primary diagnoses at Time 2. The CGIS-I improvement (ratings of 1 or 2, versus higher) for primary diagnoses were not significantly different by group (chi-square *p* > 0.05).

#### 3.2.3 Individual diagnosis CSRs

GLMMs further tested the average CSRs for individual diagnoses, including GAD, specific phobia, separation anxiety, social anxiety combined with other social fear, and fear of change combined with negative reaction to change. Specific phobia showed a marginally significant effect for Time (*F* (1, 22.67) = 4.24, *p* = 0.051), with a significant Time by Group interaction (*F* (1, 22.67) = 5.77, *p* = 0.025). Specifically, specific phobia CSRs improved for the FYF-S group (mean change from 3.37 to 1.4) while UC stayed similar over time (mean change from 2.27 to 2.22). The CSRs from combined social anxiety with other social fear also showed a significant effect for Time (*F* (1, 23.68) = 5.15, *p* = 0.033). All other models tested were non-significant.

#### 3.2.4 Mean number of DSM-5 diagnoses

**Figure 2:**
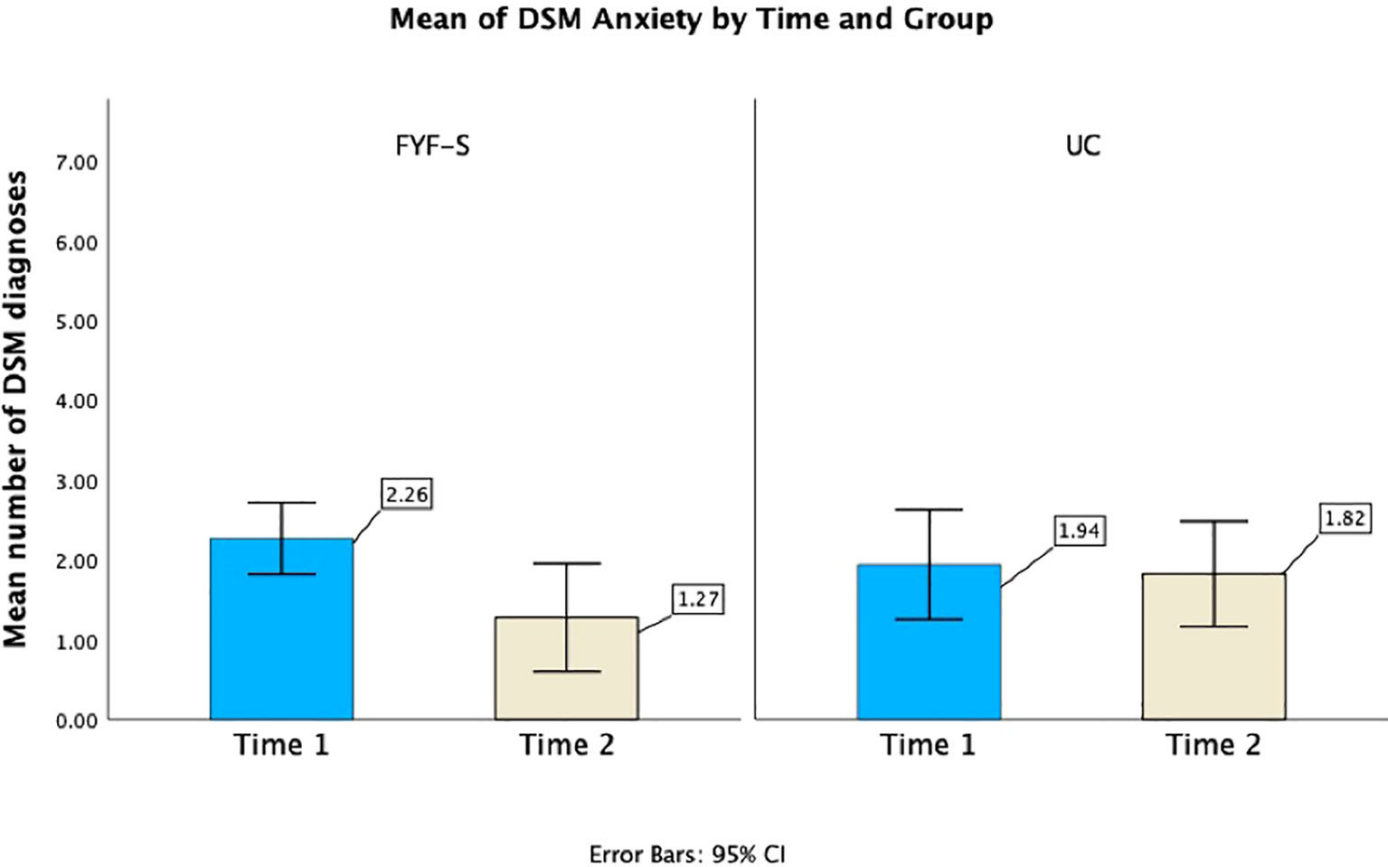
Average number of DSM-5 anxiety diagnoses for the FYF-S and UC groups at Time 1 and Time 2.

**Figure 3:**
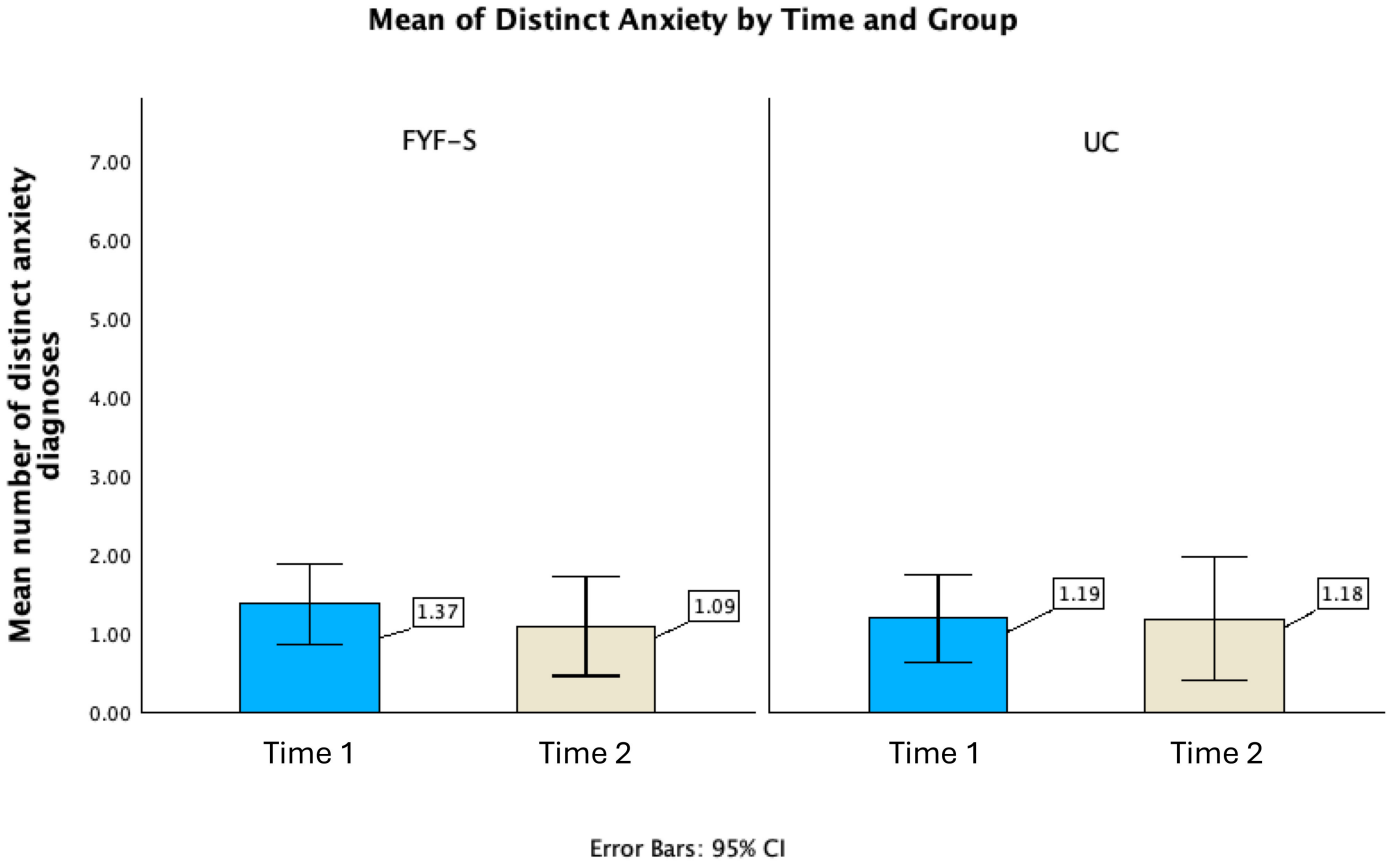
Average number of distinct anxiety diagnoses for the FYF-S and UC groups at Time 1 and Time 2.

GLMMs examining the average number of DSM-5 diagnoses (GAD, specific phobia, social anxiety, separation anxiety, and OCD) showed a significant effect for Time (*F* (1, 26.09) = 8.33, *p* = 0.008), with a marginally significant Time by Group interaction (*F* (1, 26.09) = 3.37, *p* = 0.078). Separate models for each group were explored to test individual change over time, in which FYF-S was significantly improved (*F* (1, 11.61) = 20.93, *p* < 0.001), as the mean (sd) number of DSM-5 diagnoses decreased from 2.26 (0.93) at Time 1 to 1.27 (1.01) at Time 2. In comparison, the model for UC showed no significant improvement in mean number of DSM-5 diagnoses (*p* = 0.59) from Time 1 to Time 2, 1.94 (1.29) and 1.82 (.98), respectively. GLMMs for distinct anxiety diagnoses (fear of change, negative reaction to change, other social fear, special interest fear, idiosyncratic phobia) as well as non-anxiety diagnoses were all non-significant (*p*s > 0.05; see **Figures 2**, **3**).

#### 3.2.5 CGIS-I ratings

Independent t-tests between FYF-S and UC showed significant differences in improvement for Total Anxiety Composite CGIS-I, (*t* (19) = -2.83, *p* = 0.01), in which mean (SD) FYF-S CGIS-I was 2.76 (.99) compared to a mean (SD) UC CGIS-I of 4.18 (1.30). Primary Diagnosis, Specific Phobia Composite, and Non-Anxiety Composite CGIS-I were not statistically significant between groups (*p*s > 0.05), although means for FYF-S were all lower compared to UC (see **Table 3**).

**Table 3:** Clinical Global Impressions Scale-Improvement (CGIS-I) scores for the FYF-S and UC groups.

	FYF-S	UC
	mean (SD)	mean (SD)
Primary Diagnosis	2.45 (0.93)	3.10 (0.28)
Total Anxiety Composite^1^	2.76 (0.99)	4.18 (1.30)
Specific Phobia	2.82 (1.48)	4.40 (2.56)
Non-Anxiety Composite	2.75 (0.76)	3.69 (0.76)

^1^ *p* < 0.05.

CGIS-I ratings are scored: 1 = very much improved, 4 = no change, 7 = very much worse.

## 4 Discussion

The current study is a secondary analysis from a larger cluster randomized trial examining the effectiveness of FYF-S compared to UC (28). The purpose of the present study was to further understand the nature and presentation of anxiety in a school-based sample of students with autism or suspected autism whose caregivers completed the ADIS/ASA at baseline (Time 1) and post-intervention/post-UC (Time 2). Consistent with our hypothesis, the majority of students presented with both DSM-5 and impairing distinct presentations of anxiety, although notably none of the participants presented with *only* distinct anxiety diagnoses. This finding is aligned with some previous research using the ADIS/ASA that reflects low occurrences of autistic children presenting with only distinct anxiety (5). Kerns and colleagues (52) found that autistic youth who had more significant challenges with social responsiveness (i.e., higher SRS-2 scores) presented with more distinct anxieties, thus children recruited from non-community-based settings (e.g., hospitals) who may present with more significant social communication challenges may also present with higher rates of distinct anxiety.

Within the DSM-5 anxiety diagnoses, the most common diagnosis was GAD, followed by specific phobia, social anxiety, and separation anxiety. These four DSM-5 diagnoses were also the most common in previous samples of autistic children with anxiety assessed using the ADIS/ASA (5). Importantly, the prevalence of specific phobia in this school-based sample (66%) appeared to be higher than previous samples occurring in research settings (e.g., 33% in (5)). Of the distinct anxiety presentations, negative reaction to change/fear of change comprised the vast majority of impairing concerns. The prevalence of fear of change and idiosyncratic phobias was similar to samples of school-aged autistic youth recruited in research settings, but this school-based sample had noticeably higher rates of negative reaction to change (38% compared to 7%) and other social fear (22% compared to 10%); (5)). Perhaps specific phobias, negative reaction to change, and other interfering social fears are more interfering in the school setting than home settings and children with these concerns were therefore more likely to be referred by their school providers than by parents seeking anxiety treatment in clinical settings.

ADHD was the most common non-anxiety diagnosis and comprised 57% of the sample, very similar to previous samples (5). Overall, the frequency of DSM-5 anxiety diagnoses, distinct anxiety presentations and non-anxiety diagnoses reported in this sample are generally aligned with the results of the previous research in treatment-seeking, school-aged autistic youth (35, 51), where impairing distinct anxiety has been found to co-occur with DSM-5 anxiety disorders but is less-often the only presenting anxiety concern. This is in contrast to non-treatment seeking samples, where distinct presentations are the only impairing anxiety report for 15-17% of autistic children (37).

The second aim of the study was to examine whether participants randomly assigned to FYF-S demonstrated significant improvements in anxiety compared with participants in UC, on the ADIS/ASA. Effectiveness was examined in several different ways including changes in rates of diagnoses, change in mean number of diagnoses, and change in clinical severity from Time 1 to Time 2. We also examined overall improvement in the primary diagnosis, as well as improvements in the Total Anxiety Composite, Specific Phobia Composite and Non-Anxiety Composite. Results indicated that fewer participants in FYF-S met criteria for GAD and specific phobia at Time 2 compared to participants receiving UC. In addition, participants in FYF-S demonstrated significant decreases in total number of DSM-5 anxiety diagnoses at Time 2, whereas students in UC did not display similar decreases. There were no commensurate decreases in the number of distinct anxiety diagnoses or non-anxiety diagnoses at Time 2 for either condition.

When CSRs of both DSM-5 and distinct anxiety diagnoses were examined, significant differences in CSRs were apparent for specific phobia only, indicating that FYF-S participants demonstrated significant improvements in clinical severity of specific phobia symptoms, compared with UC. Additionally, when social phobia and other social fear were combined (due to their conceptual similarities), results indicated that there was a significant effect of time but there was not a time by group interaction. Thus, severity of social fears appeared to decrease for both groups.

Finally, the CGIS-I for the Total Anxiety Composite indicated that there were significant differences in overall improvement for participants in FYF-S compared with UC. There were no significant differences in the CGIS-I for Primary Diagnosis Composite, Specific Phobia Composite or Non-Anxiety Diagnosis Composite. Of note, distinct anxiety presentations did not appear to significantly improve over time for the FYF-S condition, and although distinct anxiety diagnoses were included in the Total Anxiety Composite, it is likely that improvements in this composite may be more a reflection of improvements in DSM-5 diagnoses, rather than improvements in distinct presentations.

### 4.1 Implications

The results of the current study significantly contribute to the understanding and nature of anxiety in autistic youth. Similar to previous work, participants in this sample frequently experience impairing distinct presentations of anxiety alongside DSM-5 anxiety diagnoses (35). Although none of the participants had *only* distinct presentations of anxiety, the majority of participants had both impairing distinct anxiety and DSM-5 anxiety diagnoses, highlighting the importance of assessing for the presence of distinct anxiety presentations alongside more traditional diagnostic presentations. In addition to expected high rates of GAD, specific phobia and social anxiety, high rates of fear of change and negative reaction to change were also present. Over 17% of participants had a primary diagnosis of other social fear at Time 1. Relying only on DSM-5 criteria for characterizing anxiety may lead to an incomplete diagnostic picture for autistic youth. Of note, there was substantial overlap between GAD and fear of change, and although most participants who met criteria for fear of change also met criteria for GAD, the same was not true for participants who met criteria for GAD. This is in contrast to prior research where fears of change have been observed in autistic youth independent of GAD (37) and may reflect a tendency in this and other treatment-seeking samples (35) for autistic youth with DSM-5 anxiety, but not solely distinct anxiety presentations, to be referred for anxiety treatment. More research is needed to examine this interpretation and clarify the relationship between GAD and fear of change in autistic youth.

In the current study, although reductions in anxiety were not observed on every metric, the majority of findings do suggest that participation in FYF-S may lead to significant improvements in anxiety and are consistent with the effectiveness outcomes obtained from the larger Type 1 hybrid-effectiveness trial (27). These findings are particularly impressive given the differences between FYF and FYF-S. As noted above, FYF-S is a derivative of the original clinic-based FYF but designed for delivery in a school setting by interdisciplinary school providers. Differences across programs such as dosage, provider type and caregiver involvement are pronounced, and prior to this trial and the recently published larger trial (27), it was an open question as to whether the school-based program would be able to achieve significant reductions in anxiety similar to the clinic-based program (16, 29). FYF-S is a 12-week, 40-minute program compared with the 14-week, 90-minute clinic program. Additionally, FYF-S is delivered by interdisciplinary school providers (ISPs), while FYF is typically delivered by mental health clinicians. In FYF, caregivers attend all sessions, compared with much more limited caregiver involvement for FYF-S (two caregiver contacts). Thus, these results do appear to support the effectiveness of the school-based Facing Your Fears program.

Interestingly, significant improvements were apparent for the Total Anxiety Composite for students in FYF-S compared to students assigned to UC, but similar improvements were not apparent for the primary diagnosis. There may be several explanations for this difference. One possibility is that it is unlikely that school teams were aware of a student’s “primary” diagnosis, since the primary diagnosis is determined by caregiver interview and clinician ratings on the ADIS/ASA at Time 1 and not shared directly with ISPs. Specific anxiety targets for graded exposure practice are identified by the students in collaboration with ISPs, and rarely with caregiver input. Differences in improvement for total anxiety diagnoses compared with improvement on primary diagnosis may not only reflect differences in informants and contexts (home vs school), but also the possibility that students and ISPs may have targeted fears/worries across diagnostic categories which could be reflected in CGIS-I for total anxiety diagnoses, rather than in primary anxiety diagnosis.

With regard to specific anxiety symptoms, it appears that symptoms of GAD and specific phobia were most responsive to the intervention. On the other hand, response patterns for distinct anxieties were neither clear nor statistically significant in FYF-S, likely due to small sample sizes. Investigating treatment response of these presentations in a larger, sufficiently powered sample is warranted given their prevalence in treatment-seeking youth and the possibility that they may warrant more tailored intervention approaches.

Importantly, this is the first school-based study to use the ADIS/ASA to obtain in-depth information regarding the nature and presentation of anxiety in autistic students. Although a small subsample of a larger participant group completed the ADIS/ASA and replication with a larger sample is needed, the results do provide a richness and detail of anxiety symptoms that parent or teacher questionnaires alone cannot provide. These results also suggest that although the caregivers did not directly observe their child in school, they did notice changes in their child’s anxiety at home. These changes may have been aided by weekly caregiver handouts that they received as part of FYF-S. Thus, the results may reflect the potential benefits of the school-based intervention to generalize to the home setting as well as the ability of the ADIS/ASA to capture these changes, although more research is needed. Additionally, results suggest that in this school-based sample, the majority of participants experience both DSM-5 *and* distinct anxiety presentations. This not only underscores the critical need for school-based anxiety interventions for autistic students, but also the need for the interventions to consider creating tailored approaches to address the full presentation of anxiety (both DSM-5 and distinct anxiety) in order to be most beneficial for autistic youth.

### 4.2 Limitations

There are several important limitations in the current study, including a small sample size. Less than half of the full participant sample completed the ADIS/ASA interview at Time 1, and even fewer participants completed the ADIS/ASA at Time 2, including a low representation of families from racial/ethnic diversity, significantly limiting the generalizability of findings. Additionally, the small sample size may have also limited the ability to detect smaller effects and possibly contributed to Type II errors. Future investigations using larger samples are needed to test and establish the strength of these preliminary findings. Further, because the ADIS/ASA was offered as an optional interview, the caregivers who opted to complete this measure may have had more time for study participation; the extent to which this may have influenced other aspects of the research such as engagement in the intervention or perhaps influenced their responses to the ADIS/ASA is unknown. Larger intervention trials where the ADIS/ASA is included as a primary outcome measure are recommended. Perhaps offering greater compensation to reflect the amount of time it takes to engage in the interview could be a helpful strategy to increase caregiver participation.

Time 2 data collection may have been impacted by the COVID-19 pandemic since post-intervention data collection occurred from January 2020 through March 2020. The full extent of the pandemic in the United States was not yet known in January and February 2020, thus likely limiting the impact that the pandemic had on presentation of anxiety symptoms. It is possible that the few interviews conducted in March 2020 (*n*=5; 13.5%) may have been influenced by the pandemic, although one might argue that the ramifications of the pandemic were still unknown in March 2020. Additionally, when the ADIS/ASA is administered post-intervention caregivers are asked to reflect on their child’s anxiety symptoms over the previous six weeks, further limiting the impact of the pandemic on their responses Finally, student perspective was not considered as part of ADIS/ASA interview, limiting the report to caregivers who may be providing information based on student behavior at home, rather than at school.

### 4.3 Future research

Given the results of this study, there are several possible future research directions. Future studies may need to balance the comprehensive information that is gained from the administration of ADIS/ASA with the time it takes to administer the interview to ensure a more complete data set. As suggested above, increasing compensation for caregiver participation in the interview may be one way to increase data completion. Furthermore, in this sample interviewers asked about DSM-5 and distinct presentations of anxiety, as well as non-anxiety diagnoses such as ADHD and depression. Perhaps focusing the ADIS/ASA interview on anxiety symptoms only would shorten the interview, thus increasing data completion. In addition, this participant sample excluded students with intellectual disability (ID). The ADIS/ASA has been used with autistic youth with varied intellectual functioning in a clinical setting, and it will be important to include these students in future school-based work with the ADIS/ASA. It also may be important to consider use of the ADIS/ASA alongside teacher and child-report measures in future work [including further study of a child ASA interview; (52)]. Teacher and child reports may illustrate discrepancies in a child’s functioning across settings as well in their internal experiences and outward presentation which may enhance the sensitivity of assessments and inform treatment planning (57), but also may require a more parsimonious approach (to reduce time demands for teachers and time/emotional demands of a lengthier interview for autistic youth). Additionally, in this trial, FYF-S was compared to usual care. It would be important to conduct a comparative effectiveness trial as a next step, so that the relative merits of FYF-S could be compared against other existing school-based interventions. It had been originally hoped that a more racially and ethnically diverse sample could have been recruited for this study. Therefore, future work should make efforts to increase the diversity of the participant sample. Finally, developing specific intervention approaches that could more directly address the distinct anxiety presentations in autism would be of great benefit.

## Data Availability

The raw data supporting the conclusions of this article will be made available by the authors, without undue reservation.

## Footnotes

1 The ADIS/ASA manual indicates that fear of change is a distinct presentation of anxiety since it involves anticipation of change in routine versus negative reaction to change where anticipation is not present and rather describes an individual’s reaction to change in the moment and may not necessarily involve anxiety per se (see 50).
